# Palmitic acid content in savoury baked goods and modelled trend intake prior and post EU trans-fat regulation

**DOI:** 10.1007/s00394-025-03659-0

**Published:** 2025-04-05

**Authors:** Sotiria Kotopoulou, Georgios Marakis, Danai Papanastasiou, Stavroula Skoulika, Andreas Papaioannou, Georgios Boukouvalas, Zoe Mousia, Foteini Tzoumanika, Aggeliki Karpouza, Antonis Zampelas, Emmanuella Magriplis

**Affiliations:** 1 Hellenic Food Authority, Leoforos Kifissias 124 & Iatridou 2, Athens, 11526 Greece; 2 Department of Food Science and Human Nutrition, Laboratory of Dietetics & Quality of Life, Agricultural University of Athens, Iera Odos 75, Athens, 11855 Greece

**Keywords:** Saturated fat, Dietary exposure, Dietary intake, EU regulation

## Abstract

**Purpose:**

Palmitic acid (PA), a prevalent saturated fatty acid (SFA) in European diets, has been linked to adverse health outcomes, including cancer, cardiovascular, and neurodegenerative diseases. This study primarily aimed to assess the PA content in Savoury Baked Goods (SBGs) following the EU’s trans-fat regulation (2019/649), assuming it might be the main replacer of trans-fats, since it is widely used by the food industry due to its consistency and affordability.

**Methods:**

PA levels in SBGs from 2015 to 2021 were measured using randomly selected samples from Athens Metropolitan area. The Hellenic National Nutrition and Health Survey (HNNHS) consumption data was used to estimate PA intake.

**Results:**

An increase in PA content (in g/100 g product) was observed in most SBG types between 2015 and 2021, ranging from 3.5% in meat-containing pies to 66.7% in vegetarian pies, resulting in subsequent increased intakes. An inverse correlation trend between PA content and SBG purchase price was also observed.

**Conclusion:**

The findings underscore the importance of promoting healthier fat alternatives and further research into food reformulation practices to improve public health.

**Supplementary Information:**

The online version contains supplementary material available at 10.1007/s00394-025-03659-0.

## Introduction

The World Health Organization (WHO) in its latest guidelines on saturated fatty acids (SFAs) and trans fatty acids (TFAs) for children and adults recommends reduction of SFAs to 10% of total energy intake (TEI) or less [1], in line with other organizations and professional associations [2]. These recommendations are largely based on the positive association of SFAs with all-cause mortality risk, and coronary heart and cardiovascular diseases (CVD) incidence [3, 4]. SFAs though, comprise a wide and heterogeneous group of fatty acids. Their dietary sources and effects on human health differ [5–7], although these have not so far been adequately explored.

Palmitic acid (PA), one of the most prevailing SFAs in European diets, has been linked with adverse effects on the cardiovascular system [8]. Additionally, it has been linked to brain cell damages and neurodegenerative diseases (e.g. Alzheimer’s and Parkinson’s) [9], prostate cancer [10], and metastasis of oral carcinomas and melanoma in experimental animals [11]. Therefore, PA is of increasing public health importance, and consumption data are necessary since upper reference limits remain unknown.

A recent study in Greece which evaluated the impact of the European legislation on TFAs, reported a reduction of total TFA and industrially-produced TFAs (i-TFAs) in Savoury Baked Goods (SBGs) and a respective increase of SFAs [12]. It is known that when a substance is removed from a specific food, it is usually replaced by another that is affordable and does not alter the food composition or taste. Palmitic acid (PA) has been identified as a prominent saturated fatty acid (SFA) in many industrially produced foods and baked goods due to its consistency and the affordability of its main sources [13, 14]. Given this prevalence, it is imperative to further explore changes in the fatty acid composition of foods, emphasizing in baked goods, following product reformulation, as these changes may have potential negative implications for public health. Also, although various food composition tables include PA content, there is lack of information regarding any trend (increases or decreases) in the PA content of foods over time. In addition, standards on the nutrition quality of the fats used in the preparation of baked goods, apart from the levels of non-ruminant TFA, have not been specified at national or European level.

Therefore, the primary aim of the present study was to investigate trends in PA content in SBGs, following the introduction of Trans-fat EU Regulation (2019/649) [15]. Any changes were sought to be further quantified by conducting exposure assessment and evaluating main SBG contributors in total and by health status, using consumption data from a representative adult population sample of the Hellenic National Nutrition and Health Survey (HNNHS). The secondary aim of the study was to explore any association of PA content with the purchase price of baked goods, a parameter that is of interest during the monitoring of the effectiveness of EU Regulation on TFAs.

## Methods

### Estimation of PA content of baked goods

#### Sampling

We utilized previously unpublished PA content data which were obtained by chemical analysis of SBG samples collected in Greece, during two previously reported surveys in 2015 and 2021. Approximately twenty samples were obtained for each SBG category. Detailed sampling procedures during both sampling periods have already been reported [12, 16]. In summary, seven SBG categories were included: (1) cheese pies made with phyllo pastry (characterized by thin sheets of unleavened dough of crispy texture when baked, made from flour and water; typically prepared by brushing with olive oil or other types of vegetable oil between sheets of dough), (2) cheese pies made with shortcrust pastry, (3) cheese pies made with puff pastry, (4) bougatsa with cheese (samples collected only in 2021), (5) peinirli (i.e., pizza boat with cheese and vegetables and/or processed meat)/pizza, (6) vegetarian pies (spinach or leek pies without cheese typically made with phyllo pastry and olive or other types of vegetable oil), (7) meat containing pies (sausage or ham pies) typically made with puff pastry. During both sampling periods, all samples were collected from Attiki prefecture (Athens greater metropolitan area) by inspectors of the Hellenic Food Authority, from both major Greek bakery chains that are present within but also outside Athens metropolitan area, as well as from artisan bakeries (small businesses). Prices of purchase were also documented while on site, in 2021.

### Chemical analysis

The procedures for the estimation of PA content in SBGs in 2015 and in 2021 were identical. The chemical analyses were performed by the same scientific personnel and have previously been described in detail [12]. Briefly, each sample was homogenized and separated into 2 sub-samples that were stored at 4 °C and analyzed within 2d. The total fat content of the samples was determined by the Soxhlet method [17]. All fat samples were converted to their methyl esters (FAME) with an in-house method based on AOAC 966.06 and Roese-Gottlieb method (AOAC 905.02) [18] to avoid any possible alteration of the FAME profile [12]. The fatty acids profile was determined by Agilent 7890 A Network Gas Chromatograph (Agilent Technologies, Santa Clara, CA, USA) coupled to a flame ionization detector (FID). In the two surveys held in 2015 and 2021, two different columns were used, an Agilent (100 m×0.25 mm×0.2 μm) and a Thermo Fisher Scientific TR-FAME (50 m × 0.22 mm, 0.25 μm), respectively. Four commercial standards purchased from Supelco (Sigma) were used for the identification of the chromatograph peaks [i.e., a FAME Mix C4–C24 (*n* = 37 components), a trans-9-elaidic methyl ester standard (18:1), a linoleic acid methyl ester isomer mix (18:2) (*n* = 4) and linolenic acid methyl ester isomer mix (18:3) (*n* = 8)].

### Study design and population

The Hellenic National Nutrition and Health Survey (HNNHS) food consumption data of adult SBG consumers (*N* = 843, 56% females, 23.4% of all adult HNNHS participants) were used to estimate PA intakes using substitution models. The HNNHS surveyed a nationally representative sample of non-institutionalized, non-pregnant, and non-breastfeeding Greek adults. Food consumption was recorded through two non-consecutive 24-hour dietary recalls, 8–20 days apart, using the Automated Multiple Pass Method. Participants reported quantities using standardized grids, mounts, and verified food atlases. Detailed methodology and questionnaires from the HNNHS are available in previous publications [19]. The HNNHS study was approved by the Agricultural University of Athens Ethics Committee and the Hellenic Data Protection Authority, with all participants providing written informed consent. Consumption data, collected from September 2013 to May 2015, were combined with the results from the chemical analyses of SBG fatty acid profiles for further investigation.

### PA intake assessment

The HNNHS food consumption dataset was updated to incorporate measured PA content of SBGs sampled for the substitution models [12, 20]. In particular, the database was updated with 2015 and 2021 PA measurements on Greek often-consumed SBGs, with adding extra columns of updated nutritional information, next to the previously analyzed values of total SFA, allowing two models generated from data obtained at two different time frames to be used. Keeping SBG consumption constant (the assumption of no changes in intake was made for substitution reasons) but modifying PA content as measured, differences were calculated by comparing 2021 to 2015 assessments. Other dietary composition and intakes were also expected to remain unchanged throughout this procedure. The daily PA intakes from these products (g/day) were calculated, by multiplying the mean PA content per SBG (g in 100 g) with the product’s daily consumption (g/day) for each consumer. The PA content was adjusted based on the individual’s average energy intake as a proportion of participants’ total daily energy intake to have comparable data. Subsequently, the estimated daily intakes were summed per individual and averaged over the number of reporting days (90.6% of the participants had two 24 h recalls). The % contribution of each SBG to the overall PA intake from SBGs was additionally estimated. The SFA intakes from SBGs and in total had been previously estimated, also as % of daily total energy intakes [12, 20].

### Other parameters

Adult SBG consumers were classified into three age groups: 19–44 years, 45–59 years and ≥ 60 years. The marital status categories used were single, married/cohabiting, divorced/separated, and widowed and the educational levels were: up to 6 years, 12 years, and higher education (including colleges). Employment status was grouped into unemployed, employed, and pension. Physical activity level was defined according to calculation standards as low, sedentary, moderate and high based on results from the International Physical Activity Questionnaire IPAQ [21]. Smoking status was also assessed and subjects were classified as current- ex- or non- smokers. Current smokers were those that had smoked at least 100 cigarettes in their lifetime and reported smoking daily or on some days at the interview, ex-smokers had smoked at least 100 cigarettes but not in the past 30 days and never smokers had never smoked at least 100 cigarettes in total [22]. Body Mass Index (BMI; kg/m^2^) was calculated using participants’ weight and height. According to WHO, weight status was categorized as healthy (BMI < 25 kg/m^2^), overweight (25 ≤ BMI < 30 kg/m^2^), or obese (BMI ≥ 30 kg/m^2^) [23]. Disease status for hypertension, Type 2 Diabetes, hyperlipidemia, and/or CVD was assessed based on the following characteristics: (a) a self-reported positive response, (b) a documented diagnosis by a medical professional and current use of respective medications (as confirmed by the trained interviewer), or (c) an abnormal profile as determined by blood sampling (lipids, fasting glucose) or average blood pressure over 140/90 mm Hg [19]. Participants with CVD included individuals with arrhythmia, coronary heart disease, myocardial infarction, angina, heart failure, or a history of stroke, whereas those with no hypertension, dyslipidemia, or any other CVD-related conditions were categorized as healthy for further analysis. Differences between the total study sample (*N* = 843) and sub-totals per variables examined (e.g. sex, age, etc.), as presented in Tables and Figures, are attributed to missing data.

### Statistical analysis

Baseline variables and PA intake from SBGs were stratified according to total SFA intakes (% of total daily energy intake) to identify statistically significant differences between intakes (p-value, p-trend value). Contribution of different SBGs to total PA derived energy intake was also estimated by age group and health status, as well as the correlation between mean PA content and purchase price, all using 2021 composition data. Means (Standard Deviations, SD) were used to describe normally distributed continuous variables and medians (25th, 75th percentiles) were used for skewed distributions. Categorical variables were expressed as frequencies, and between group distribution differences were examined using chi square for proportions. ANOVA or Kruskal Wallis rank sum were used for continuous data, depending on distribution. Pearson’s function was used to test the correlation between price of purchase and PA content of each type of SBG product. Level of significance was set at 5%. Statistical analysis was done using STATA 13.0 (Texas ltd, Texas, USA).

## Results

Table 1 shows measured total SFA and PA levels per SBG sampled in 2021 compared to 2015 (mean difference depicted for each). The PA levels of SBGs tracked SFA content trends in all SBGs except meat-containing pies, in which PA increased (+ 3.5%) although SFA decreased (-0.9%). Overall, PA content increased in all SBGs other to cheese pies with phyllo pastry.

**Table 1:** Contents of total fat^a^ (g), palmitic acid (g/100 g fat), SFA^b^ (g) and palmitic acid (g/100 g product as purchased) in different types of non-prepacked SBGs^c^ (mean and standard deviation) from bakeries in Greece, in 2015 and 2021

Type of product	Total fat^a^ g(2015)	Palmitic acid, g/100 g fat (2015)	Total fat^a^ g(2021)	Palmitic acid, g/100 g fat (2021)	SFA^b^ g(2015)	SFA^b^ g(2021)	%SFA^b^ change	Palmitic acid, g/100 g product (2015)	Palmitic acid, g/100 g product (2021)	%Palmitic acid change
Cheese pies with **phyllo** pastry	19.5 (4.5)	27.1 (9.7)	19.2 (5.0)	22.5 (7.6)	7.7 (3.6)	7.3 (3.6)	-5.2	5.7 (2.8)	4.6 (2.7)	-19.3
Cheese pies with **shortcrust** pastries	19.7 (4.5)	34.9 (3.5)	25.1 (3.8)	34.5 (7.5)	9.6 (2.1)	12.1 (2.5)	26.0	6.9 (1.9)	8.8 (2.5)	27.5
Cheese pies with **puff** pastry	22.5 (2.3)	41.9 (3.1)	25.7 (3.0)	44.3 (2.7)	12.4 (1.3)	14.1 (1.7)	13.7	9.4 (1.0)	11.3 (1.3)	20.2
**Bougatsa** with cheese^d^	-	-	17.7 (3.8)	31.6 (4.6)		8.1(2.2)	NA		5.7 (1.8)	NA
**Pizza/Peinirli** ^e^	11.7 (1.4)	27.8 (2.2)	12.5 (2.3)	29.4 (4.1)	6.3 (0.8)	6.8 (1.9)	7.9	3.2 (0.3)	3.7 (1.0)	15.6
**Vegetarian** pies (e.g., spinach or leek pie)	11.4 (0.5)	15.6 (6.9)	17.6 (4.0)	17.4 (11.4)	2.3 (0.8)	4.0 (2.2)	73.9	1.8 (0.8)	3.0 (2.0)	66.7
**Meat-containing** pies (e.g., sausage pies, ham pie)^f^	22.2 (2.0)	39.0 (2.7)	23.1 (4.7)	37.3 (9.5)	11.2 (0.5)	11.1 (3.5)	-0.9	8.6 (0.9)	8.9 (3.1)	3.5

^a^ Total fat Data reproduced: from reference [12] with permission

^b^ SFA: Saturated fatty acids/Data reproduced from reference [12] with permission

^c^ SBG: Savory baked goods;

^d^ Bougatsa with cheese samples were collected only in 2021

^e^ In 2015, pizza slice samples from bakeries were collected, whereas in 2021 pizza boat (peinirli) samples from bakeries were collected

^f^ In 2015, only sausage pies were collected (*N* = 5), whereas in 2021, sausage pies (*N* = 16), cheese and ham pies (*N* = 3), and cooked beef in tomato sauce pies (*N* = 1) were collected

^g^ Results account for all SBGs analyzed, accounting for content variation (in each type). For instance, for meat-containing pies the median (p25, p75) values for palmitic acid (g/in 100 g product) are 8.7 (8.5, 8.8) in 2015 and 9.6 (8.5, 10.9) in 2021 respectively

Consumption data of PA and SFA intake on SBG consumers are depicted in Table 2, in total and by total %SFA tertiles. In 2021, adult SBG consumers had a mean total SFA intake of 14.8% of daily energy intake, ranging from 10.1% in the first tertile to 19.7% in the third tertile, compared to 14.1% in 2015 (10.0-18.4). PA intake from SBGs was 2.6% of daily energy intake, ranging from 1.9% in the first tertile to 4.0% in the third tertile, with a significant increasing trend (*p* < 0.001), compared to 2.3% in 2015 (1.6–3.5%). The baseline sociodemographic and other personal characteristics of adult SBG consumers, including health status parameters, are presented in Supplementary Table S1.

**Table 2:** Fatty acid intakes for adult SBG^a^ consumers (≥ 19 years) overall and by total SFA^b^ intakes, adjusted for individual mean energy intake using substitution models^c^

Variables	Population N=849; SFA intake, %energy, mean (sd^d^): 14.8 (4.5)	Total SFA tertiles of adult consumers (2021)			p for differences		p-trend	
		1st Tertile N=284; SFA intake, %energy, mean (sd): 10.1 (1.9)	2nd Tertile N=283; SFA intake, %energy, mean (sd): 14.6 (1.1)	3rd Tertile N=282; SFA intake, %energy, mean (sd): 19.7 (3.00)				
**Palmitic Acid from savoury bakery products 2015**,** %energy**,** median (25th**,** 75th percentile)**	**2.3** **(1.2**,** 4.3)**	**1.6** **(0.9**,** 2.3)**	**2.5** **(1.2**,** 4.1)**	**3.5** **(1.7**,** 6.0)**	**< 0.001**		**< 0.001**	
**Palmitic Acid from savoury bakery products 2021**,** %energy**,** median (25th**,** 75th percentile)**	**2.6** **(1.5**,** 4.7)**	**1.9** **(1.1**,** 3.1)**	**2.6** **(1.4**,** 4.4)**	**4.0** **(1.8**,** 6.8)**	**< 0.001**		**< 0.001**	
Total SFA intake 2015, %energy, median (25th, 75th percentile)	14.1 (11.1, 17.1)	10.0 (8.3, 11.2)	14.1 (13.2, 15.1)	18.4 (17.1, 20.5)	**< 0.001**		**< 0.001**	
SFA intake from savoury bakery products 2015, %energy, median (25th, 75th percentile)	3.1 (1.6, 5.9)	2.2 (1.2, 3.7)	3.3 (1.6, 5.6)	4.7 (2.3, 5.8)	**< 0.001**		**< 0.001**	
SFA intake from savoury bakery products 2021, %energy, median (25th, 75th percentile)	3.6 (2.0, 6.3)	2.7 (1.6, 4.2)	3.8 (2.0, 5.8)	5.2 (2.7, 8.5)	**< 0.001**		**< 0.001**	

^a^ SBG: Savoury baked goods

^b^ SFA: Saturated Fatty Acids

^c^ Substitution models: measured content in savoury baked goods in 2021 replaced those already used in previous research during the Hellenic National Nutrition and Health Survey study years (2015) to evaluate palmitic acid intake amount post Regulation (EU) 2019/649, assuming intakes of other foods remained constant

^d^ sd: standard deviation

**Fig. 1:**
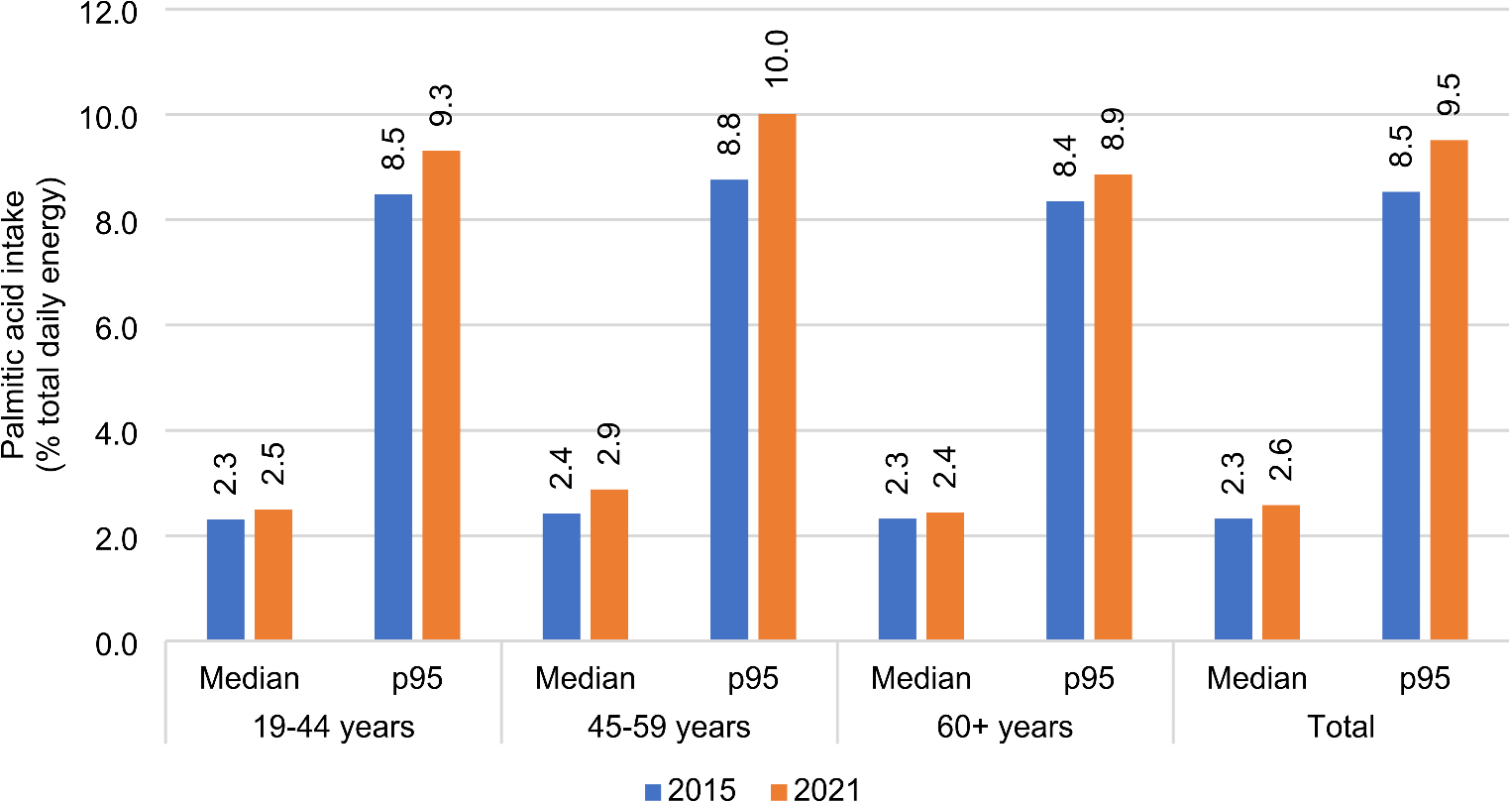
Palmitic acid intakes from all SBGs^a^, as a percentage of daily energy intake at median and p95^b^ values in total and by age group^c^ in 2015 and 2021, using substitution models^d^. ^a^. SBGs: Savoury baked goods. ^b^. p95: 95^th^ percentile. ^c^. Distribution of PA intake significantly differed between 2015 and 2021 in all age groups (*p* < 0.001). ^d^. Substitution models: measured content in savoury baked goods in 2021 replaced those already used in previous research during the Hellenic National Nutrition and Health Survey study years (2015) to evaluate palmitic acid intake amount post Regulation (EU) 2019/649, assuming intakes of other foods remained constant

Specific differences in PA intakes from SBGs in total and by age group at median and 95th percentile (p95, high consumers), in 2015 and 2021 respectively, are shown in Fig. 1. Although the median PA intake from SBGs increased from 2.3% of total energy intake in 2015 to 2.6% of total energy intake in 2021 (13% increase, *p* < 0.001), the greatest difference was observed among the high consumers in the age group 45–59 years (p95, 13.6% increase).

**Fig. 2:**
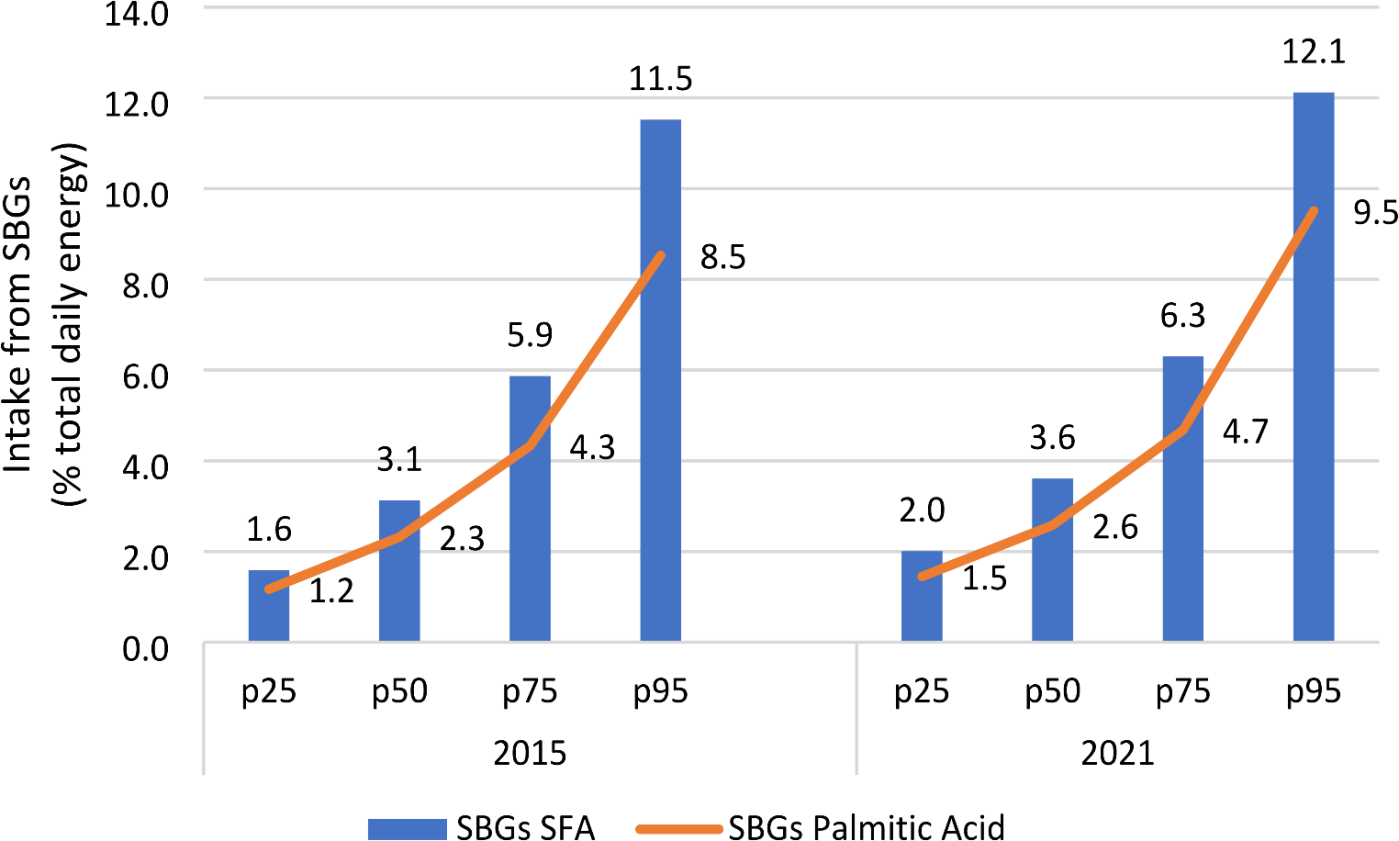
Distribution of median SFA^a^ and Palmitic Acid intakes from SBGs^b^ as a percentage of daily total energy intake for consumers of SBGs in 2015 and 2021, using substitution models^c^. ^a^. SFA: Saturated Fatty Acids. ^b^. SBG: Savoury baked goods. ^c^. Substitution models: measured content in savoury baked goods in 2021 replaced those already used in previous research during the Hellenic National Nutrition and Health Survey study years (2015) to evaluate palmitic acid intake amount post Regulation (EU) 2019/649, assuming intakes of other foods remained constant

Figure 2 illustrates the distribution of median SFA and PA intakes from SBGs, showing a proportionate increase in both SFA and PA intake.

**Fig. 3:**
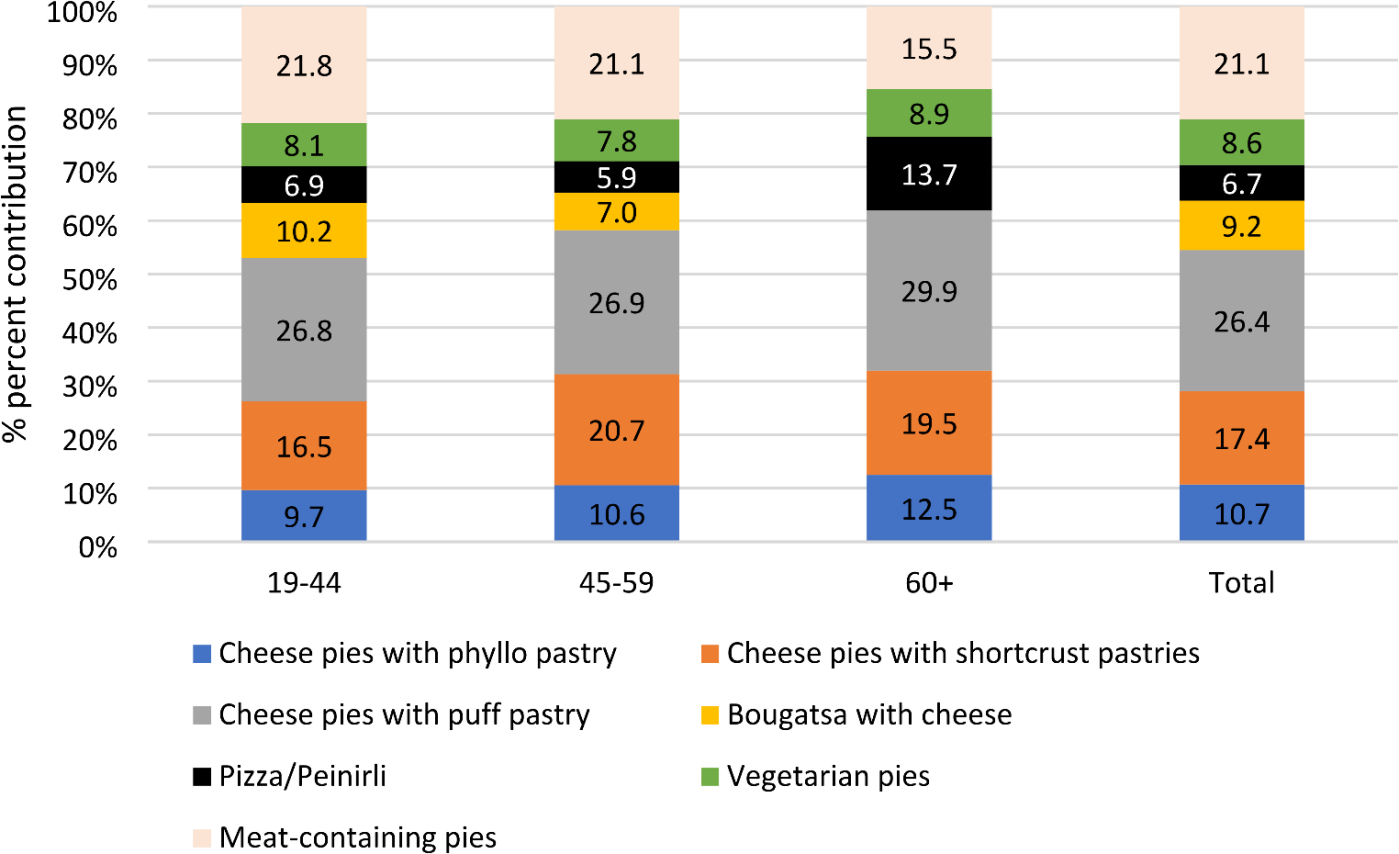
Contribution of different SBGs^a^ to total palmitic acid derived energy intake, by age group and in total in 2021, based on substitution models^b^. ^a^. SBGs: Savoury baked goods. ^b^. Substitution models: measured content in savoury baked goods in 2021 replaced those already used in previous research during the Hellenic National Nutrition and Health Survey study years (2015) to evaluate palmitic acid intake amount post Regulation (EU) 2019/649, assuming intakes of other foods remained constant

Supplementary Figure S1 depicts the median intakes of each type of non-prepackaged SBG in 2015 and 2021, expressed as percentages of daily total energy intakes and it demonstrates that the greater increase in palmitic acid intake was observed from the consumption of cheese pies with shortcrust pastry (27.6%), followed by cheese pies with puff pastry (20.7%). The only SBG for which the consumption led to a decrease in PA intake between 2015 and 2021 was cheese pies with phyllo pastry (-19.4%). Figure 3 presents the contribution of each SBG type to the total PA derived energy intake in 2021 by age group and overall. In general, the primary sources of PA derived from SBGs are cheese pies with puff pastry, meat-containing pies, and cheese pies with shortcrust pastry, as also observed in Figure S1, with varying percentages among age groups.

**Fig. 4:**
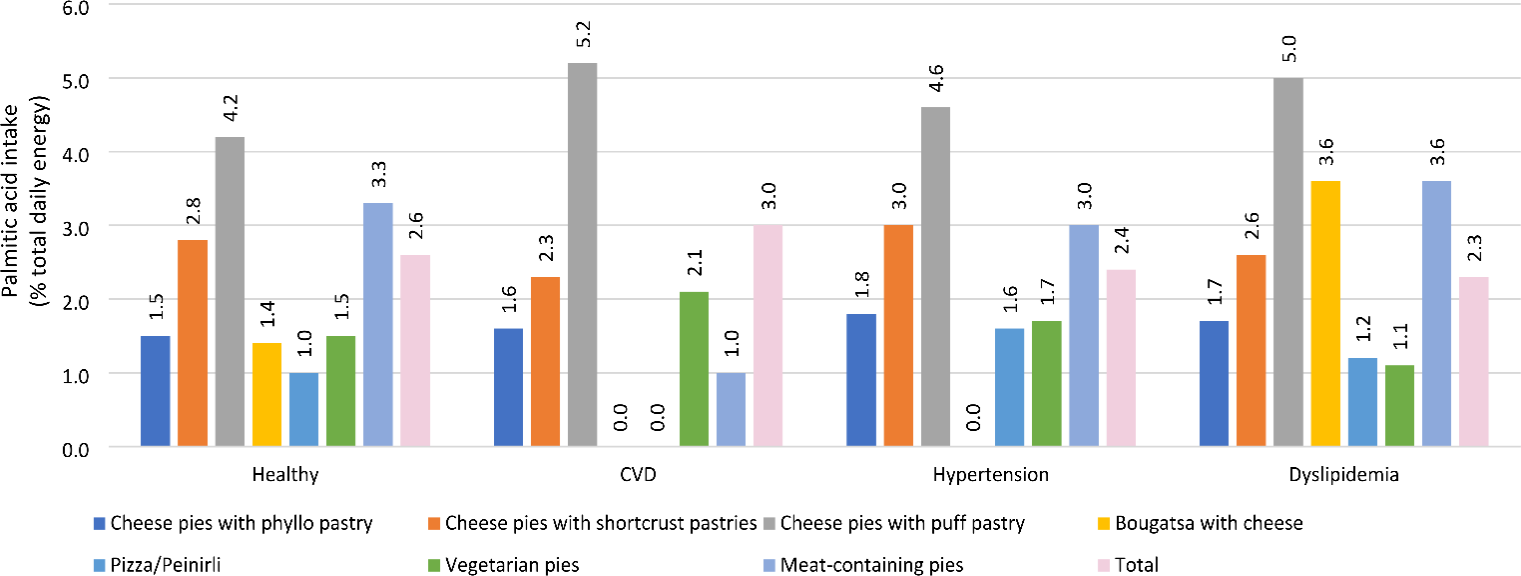
Contribution of different SBGs^a^ to total palmitic acid derived energy intake in Greek adult participants categorized as healthy^b^, with CVD^c^, with hypertension^c^ and with dyslipidemia^e^ (using 2021 composition data), based on substitution models^f^. ^a^. SBGs: Savoury baked goods. ^b^. Healthy: Categorized with no hypertension or dyslipidemia or any other CVD-related condition. ^c^. CVD: Cardiovascular Diseases; With CVD: Categorized with arrhythmia, coronary heart disease, myocardial infarction, angina, heart failure, history of stroke (one or more). ^d^. Hypertension: Defined as average blood pressure over 140/90 mm Hg or on antihypertensive medication. ^e^. Dyslipidemia: If they exhibited any of the following characteristics: (**a**) a self-reported high cholesterol and/or triglyceride level, (**b**) a documented diagnosis by a medical professional and current use of antilipidemic medications (as confirmed by the trained interviewer), or (**c**) an abnormal lipid profile as determined by blood sampling. ^f^. Substitution models: measured content in savoury baked goods in 2021 replaced those already used in previous research during the Hellenic National Nutrition and Health Survey study years (2015) to evaluate palmitic acid intake amount post Regulation (EU) 2019/649, assuming intakes of other foods remained constant

**Fig. 5:**
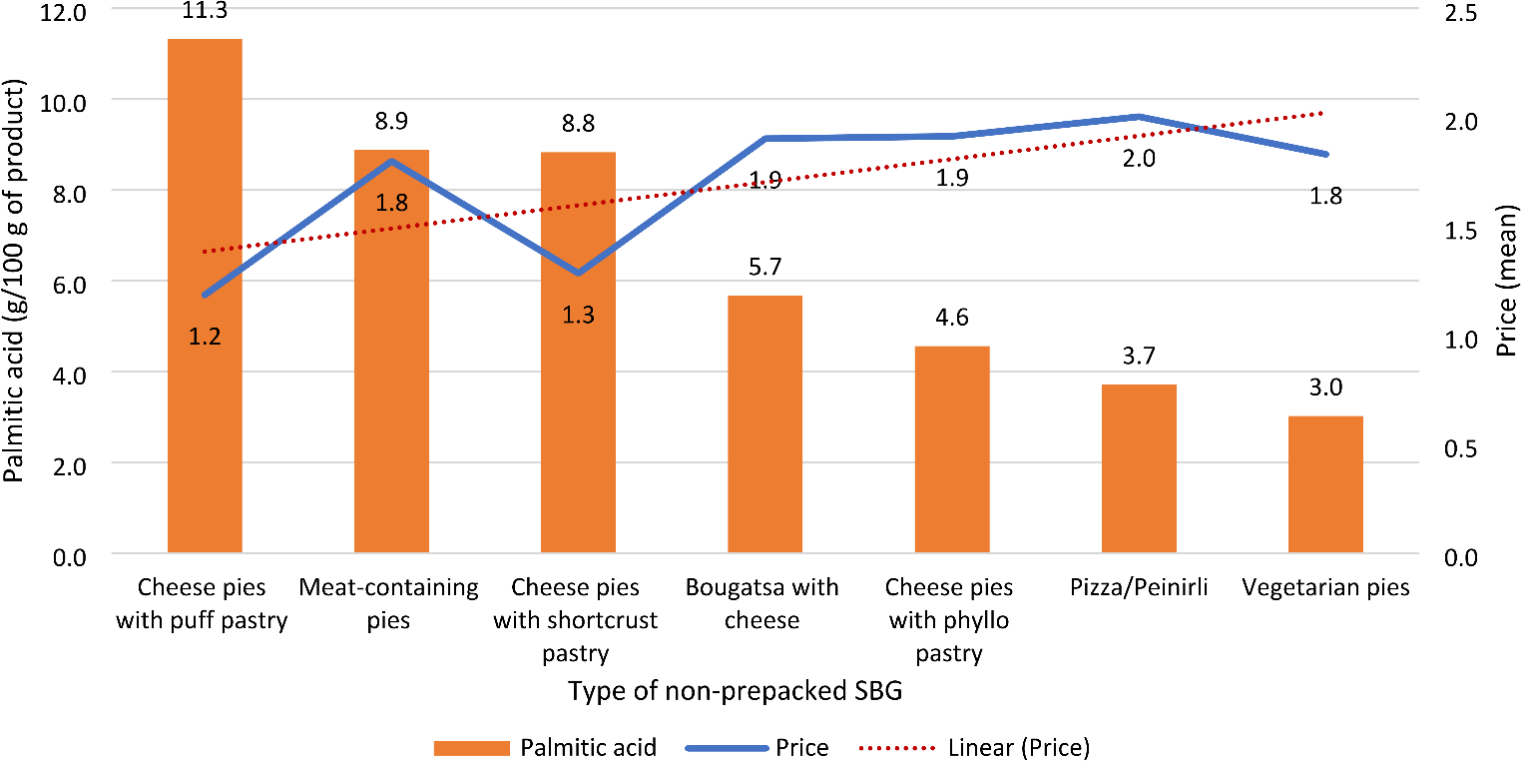
Mean price of purchase (€) and mean palmitic acid content (g/100 g of product) by type of non-prepacked SBG^a^. ^a^ SBG: Savoury baked goods

Figure 4 demonstrates that individuals classified as having CVD, as well as those diagnosed with hypertension or dyslipidemia, exhibit a higher intake of PA from cheese pies with puff pastry. Finally, the analysis revealed a significant correlation between SBG purchase prices and their PA content (*p* < 0.001) in the 2021 sampling for which prices were available. Figure 5 illustrates this linear correlation between mean SBG prices per SBG type and average palmitic acid content (g/100 g of product), with exceptions to this trend for meat-containing pies and vegetarian pies.

## Discussion

To the best of our knowledge, this is the first study that investigated trends of PA content in specific foods, namely SBGs and their subsequent intake, due to their associated potential public health implications. Our focus on PA reflects its potential role as a predominant replacement for i-TFA in industrial fats, due to its consistency and affordability, despite its high atherogenicity [8–11, 13, 14]. The analysis on fatty acid profile of SBGs indicated that, following the EU Regulation 2019/649, PA content increased in all non-prepackaged SBGs except for cheese pies with phyllo pastry. PA changes between 2015 and 2021 paralleled SFA trends seen in SBGs in most of the products and were found to be a key determinant of the observed SFA increases in 2021 compared to 2015, leading to an overall increased intake in all consumption levels, and flagging high consumers. The main SBG contributor was cheese pie with puff pastry irrespective of age and health status. While manufacturers have worked to eliminate i-TFA following EU regulations [12], this analysis suggests that the resulting increase in PA may negate the potential public health benefits of i-TFA reduction, especially for those with chronic diseases.

Different SFAs have different impacts on health [5–7]. Therefore, it is pertinent when food reformulation leads to increments in total SFA, to identify which ones are mostly increased. PA is a type of SFA for which there is evidence for adverse health effects [9, 24–26], affecting particularly CVD heath differently compared to other SFA [27, 28], but neither the European Food Safety Authority (EFSA) nor any other scientific body though has established a health-based guidance value (HBGV). In the absence of a dietary reference value for PA, monitoring the presence and trends of this fatty acid in processed foods and body tissue levels is warranted for future risk assessments. Our analysis of PA intakes across SBGs following EU trans-fat regulation provides valuable insights for developing targeted dietary public health interventions, in terms of total consumption. More specifically policies that promote food reformulation does not necessarily make a food healthier, as in the case of SBG’s, an important aspect particularly for populations with or at risk for CVD.

While the changes in SFA and PA levels trend similarly, their different magnitudes (e.g. 13.7% vs. 20.2% increase in puff pastry pies, respectively) suggest changes not only in total fat content but also in type of fat replacement. Furthermore, the reformulations that have probably taken place can result in increased absolute SFA and PA intake, even if proportional SFA levels within the fat matrix remained relatively stable. Obviously, in the context of food reformulation, a relatively stable contribution of SFA to the total fat content in two sampling periods, does not necessarily mean that the contribution of individual SFA to the total fat content are also stable. The increments seen in PA following EU Regulation 2019/649 and reformulation activities, suggest greater use of palm oil for the preparation of these products. Palm oil is considered to be a rich source of PA [29], consisting of approximately 45% PA [30, 31]. The recent FAO report has shown that global palm oil production has increased by 241% from 2000 to 2020 [32]. Although some amounts of palm oil are used for non-food purposes (e.g. biofuels, candles, cosmetics), market trends have shown that manufacturers are switching from hydrogenated fats to TFA-free alternatives produced from palm oil, for food product reformulations [33]. In particular, palm stearin can provide the solid fat functionality without the use of hydrogenation [31] and is particularly desirable in formulations of pastry margarines and shortenings [33]. The oxidative stability and functional properties of this oil, the guaranteed supply and its low cost have made it a preferred raw material for food processing companies [34], particularly baked goods [30].

In some EU countries, governments are working to improve the food composition of processed foods, recommending substitution of palm oil with other healthier oils [34]. For example, according to the latest Nordic nutrition recommendations in 2023, palm oil use is not recommended [35]. This recommendation is based not only on the LDL-cholesterol-increasing potential of this oil compared with oils rich in MUFAs and PUFAs but also because it is a major driver for deforestation and has the highest carbon and biodiversity impacts of all vegetable oils. In the latest Greek dietary guidelines published in 2014, olive oil is the recommended source of fat in the diet. Cheese pies with phyllo pastry are usually prepared with olive oil [12]– typically low in SFA and PA [36]– added between dough sheets; hence no increase in PA was anticipated in this type of products. Nevertheless, there is no specific guidance to substitute food sources high in PA and/or palm oil with those higher in unsaturated fatty acids, possibly since there has been no indication of such an increase in PA previously. Subsequently this study found that PA intake from specific baked goods increased in 2021 across all age groups with the increase affecting all consumers but especially high consumers. This trend was observed in both healthy adults and those with cardiovascular conditions. It is imperative to advise limiting puff pastry consumption, particularly for those with cardiometabolic diseases, until more drastic public health actions are placed, such as specifics on the fat replacer.

Prices are an important determinant of food choices [37], and consequently an important aspect in nutrition-related policies. A trend of inverse association was observed between the price of SBGs purchased and their PA content in the 2021 sampling. This further supports the notion of an increased use of palm oil for the preparation of these products, since palm oil is also considered the cheapest oil in the world and the most marketable one worldwide [29]. Any future food tax policy in Greece needs to be carefully designed to take into account the cost of raw materials, encouraging the use of healthier fats in food manufacturing with low carbon impact, while ensuring food adequacy and security among the most vulnerable segments of population.

### Limitations

An increased use of palm oil/palm oil blends has been hypothesized following non-prepackaged SBGs reformulation. However, the absence of ingredients list in non-labelled foods renders difficult any robust conclusions to be drawn regarding the exact type of fat/oil that replaced the partially hydrogenated fats. Another limitation of this study is the lack of recent food consumption data from a nationally representative sample of SBG consumers. For this reason, substitution models were employed using data collected from HNNHS, based on measurements performed in 2021. Lastly, we didn’t analyze other processed foods which would otherwise enable us to reach more robust conclusions about the reformulation practices in other food categories following EU Regulation 2019/649.

## Conclusion

The study represents one of the first to explore trends in content and intake of PA before and after the EU Regulation 2019/649, which aimed to reduce industrial trans-fatty acids (i-TFA) in foods, revealing a potential public health consequence. While successful in lowering i-TFA in SBGs, subsequent to the legislation, an increase in PA content in most of these products has been observed. This shift, likely due to increased use of palm oil as a replacement for partially hydrogenated fats, potentially mitigates the intended public health benefits of the act, for the general population but especially for high-risk individuals with chronic diseases. An inverse association was observed between the price of SBGs and their PA content. The findings highlight the need for a more comprehensive approach to food reformulation that considers overall nutritional impact, encourages the use of healthier fat alternatives and not only obligatory removal, while simultaneously addressing food pricing policies. Future policy actions should address this complex issue through updated dietary guidelines, and continued monitoring of fatty acid profiles in more processed foods to inform risk assessments and policy decisions.

Supplementary Information: The online version contains supplementary material available at………………………: Table S1: Baseline characteristics for adult SBG consumers (≥19 years) overall and by total SFA intakes, using substitution models. Figure S1: Palmitic acid intakes, as a percentage of daily total energy intake at median (p25, p75) by SBG product in 2015 and 2021, using substitution models.

## Electronic Supplementary Material

Below is the link to the electronic supplementary material.

Supplementary Material

## Data Availability

Data described in the manuscript will be made available upon request, pending application and approval.
