# Supplementary material for: Palmitic acid content in savoury baked goods and modelled trend intake prior and post EU trans-fat regulation
Source: Eur J Nutr. 2025 Apr 5;64(4):147. doi: 10.1007/s00394-025-03659-0 (PMC11972189; doi:10.1007/s00394-025-03659-0)
Supplement: Supplementary file 1 — Supplementary Material [file 394_2025_3659_MOESM1_ESM.docx]

# **European Journal of Nutrition**

# **Supplementary Information**

# **Palmitic Acid content in Savoury Baked Goods and modelled trend intake prior and post EU trans-fat Regulation**

Sotiria Kotopoulou^1^, Georgios Marakis^1^, Danai Papanastasiou^1^, Stavroula Skoulika^1^, Andreas Papaioannou^1^, Georgios Boukouvalas^1^, Zoe Mousia^1^, Foteini Tzoumanika^1^, Aggeliki Karpouza^1^, Antonis Zampelas^1,2^, Emmanuella Magriplis^2^*

^1^Hellenic Food Authority, Leoforos Kifissias 124 & Iatridou 2, 11526 Athens, Greece

^2^Agricultural University of Athens, Department of Food Science and Human Nutrition, Laboratory of Dietetics & Quality of Life, Iera Odos 75, 11855 Athens, Greece

***** Correspondence: [emagriplis@aua.gr](mailto:emagriplis@aua.gr)

**Table S1:** Baseline characteristics for adult SBG^a^ consumers (≥19 years) overall and by total SFA^b^ intakes, using substitution models^c^.

| **Variables** |  | **Total SFA tertiles of adult consumers (2021)** | | |  |  |
| --- | --- | --- | --- | --- | --- | --- |
|  | **Population N=849; SFA intake, %energy, mean (sd^d^): 14.8 (4.5)** | **1st Tertile N=284; SFA intake, %energy, mean (sd): 10.1 (1.9)** | **2nd Tertile N=283; SFA intake, %energy, mean (sd): 14.6 (1.1)** | **3rd Tertile N=282; SFA intake, %energy, mean (sd): 19.7 (3.0)** | *p* for differences | *p*-trend |
| Age(years), median (25^th^, 75^th^ percentile) | 36 (26, 53) | 38 (27.5, 52) | 36 (26, 51) | 34 (25, 54) | 0.153 | 0.058 |
| Age groups, n (%) |  |  |  |  | **<0.05** | 0.846 |
| 19-44 years | 554 (65.3) | 178 (62.7) | 190 (67.1) | 186 (66.0) |  |  |
| 45-59 years | 160 (18.8) | 68 (23.9) | 50 (17.7) | 42 (14.9) |  |  |
| 60+ years | 135 (15.9) | 38 (13.4) | 43 (15.2) | 54 (19.1) |  |  |
| Sex, n (%) |  |  |  |  | 0.931 | 0.814 |
| Females | 473 (55.8) | 161 (56.7) | 156 (55.1) | 156 (55.7) |  |  |
| Males | 374 (44.2) | 123 (43.3) | 127 (44.9) | 124 (44.3) |  |  |
| Total energy intake (kcal/day), mean(sd) | 2169.4 (972.8) | 2266.4(1104. 9) | 2087.4 (856.5) | 2154.1 (934.7) | 0.086 | 0.621 |
| BMI ^e^ (kg/m^2)^)^2^, mean(sd) | 25.28 (4.6) | 25.40 (4.4) | 25.01 (4.8) | 25.42 (4.8) | 0.504 | 0.668 |
| BMI categories^3^, n (%) |  |  |  |  | 0.567 | 0.359 |
| Healthy weight | 449 (54.5) | 143 (51.8) | 159 (57.2) | 147 (54.4) |  |  |
| Overweight | 243 (29.5) | 90 (32.6) | 78 (28.1) | 75 (27.8) |  |  |
| Obese | 132 (16.0) | 43 (15.6) | 41 (14.7) | 48 (17.8) |  |  |
| Marital status, n (%) |  |  |  |  | **<0.05** | 0.279 |
| Single | 417 (50.0) | 129 (45.6) | 140 (50.4) | 148 (54.1) |  |  |
| Married/Cohabiting | 351 (42.1) | 136 (48.1) | 107 (38.5) | 108 (39.6) |  |  |
| Divorced/Separated | 34 (4.1) | 6 (2.1) | 18 (6.5) | 10 (3.7) |  |  |
| Widowed | 32 (3.8) | 12 (4.2) | 13 (4.7) | 7 (2.6) |  |  |
| Education level, n (%) |  |  |  |  | 0.335 | 0.4900 |
| Up to 6years of school | 67 (7.9) | 20 (7.0) | 19 (6.7) | 28 (10.0) |  |  |
| 12 years of school | 308 (36.5) | 109 (38.4) | 95 (33.7) | 104 (37.3) |  |  |
| Higher education (including colleges) | 470 (55.6) | 155 (54.6) | 168 (59.6) | 147 (52.7) |  |  |
| Employment status, n (%) |  |  |  |  | 0.444 | 0.101 |
| Unemployed | 237 (28.1) | 68 (23.9) | 84 (29.8) | 85 (30.6) |  |  |
| Employed | 483 (57.2) | 173 (60.9) | 157 (55.7) | 153 (55.0) |  |  |
| Pension | 124 (14.7) | 43 (15.2) | 41 (14.5) | 40 (14.4) |  |  |
| Physical activity status, n (%) |  |  |  |  | 0.750 | 0.827 |
| Low | 109 (13.3) | 32 (11.4) | 44 (16.3) | 33 (12.3) |  |  |
| Moderate | 325 (39.6) | 113 (40.7) | 106 (39.2) | 106 (39.5) |  |  |
| Sedentary | 66 (8.1) | 22 (7.9) | 21 (7.8) | 23 (8.6 |  |  |
| High | 318 (38.88) | 113 (40.36) | 99 (36.7) | 106 (39.6) |  |  |
| Smoking status, n (%) |  |  |  |  | **<0.05** | **<0.05** |
| Never smoked | 434 (51.5) | 134 (47.3) | 142 (50.4) | 158 (56.8) |  |  |
| Current smoker | 306 (36.3) | 112 (39.6) | 112 (39.7) | 82 (29.5) |  |  |
| Ex smoker | 103 (12.2) | 37 (13.1) | 28 (9.9) | 38 (13.7) |  |  |
| Meddiet score, mean(sd) | 27.60 (5.9) | 28.93 (5.8) | 27.49 (6.1) | 26.36 (5.610) | **<0.001** | **<0.001** |
| Meddiet category, n(%) |  |  |  |  | 0.113 | **<0.05** |
| MD^f^<23 | 146 (18.3) | 41 (15.4) | 47 (17.5) | 58 (22.2) |  |  |
| MD>=23 | 651 (81.7) | 226 (84.6) | 222 (82.5) | 203 (77.8) |  |  |
| Sodium intake, n(%) |  |  |  |  | **<0.05** | **<0.05** |
| <1500 | 107 (13.4) | 43 (16.1) | 26 (9.7) | 38 (14.6) |  |  |
| >=1500 & <2300 | 400 (50.2) | 137 (51.3) | 149 (55.4) | 114 (43.7) |  |  |
| =<2300 | 290 (36.4) | 87 (32.6) | 94(34.9) | 109 (41.7) |  |  |
| Systolic BP^g^ in mmHg, mean (sd) | 119.1 (13.7) | 118.4 (12.3) | 118.9 (13.0) | 120.2 (15.8) | 0.680 | 0.662 |
| Diastolic BP in mmHg, mean (sd) | 72.8 (10.6) | 72.2 (11.3) | 72.13 (10.0) | 72.9 (10.4) | 0.882 | 0.804 |
| Hypertension, n (%) |  |  |  |  | 0.091 | **<0.05** |
| No | 732 (88.0) | 254 (91.0) | 246 (87.9) | 232 (85.0) |  |  |
| Yes | 100 (12.0) | 25 (9.0) | 34 (12.1) | 41 (15.0) |  |  |
| Dyslipidemia (total), n (%) |  |  |  |  | 0.703 | 0.886 |
| No | 539 (69.1) | 185 (70.3) | 176 (67.2) | 178 (69.8) |  |  |
| Yes | 241 (30.9) | 78 (29.7) | 86 (32.8) | 77 (30.2) |  |  |
| Cardiovascular disease, n (%) |  |  |  |  | 0.326 | 0.1418 |
| No | 797 (97.2) | 268 (96.1) | 267 (97.4) | 262 (98.14) |  |  |
| Yes | 23 (2.8) | 11 (3.9) | 7 (2.5) | 5 (1.9) |  |  |

1. SBG: Savory baked goods
2. SFA: Saturated Fatty Acids
3. Substitution models: measured content in savory baked goods in 2021 replaced those already used in previous research during the Hellenic National Nutrition and Health Survey study years (2015) to evaluate palmitic acid intake amount post Regulation (EU) 2019/649, assuming intakes of other foods remained constant.
4. sd: standard deviation
5. BMI: Body Mass Index; 2Body Mass Index (BMI; kg/m2) was calculated using participants' weight and height; 3Weight status was categorized as healthy (BMI < 25 kg/m2), overweight (25 ≤ BMI < 30 kg/m2), and obese (BMI ≥ 30 kg/m2)
6. MD: MedDiet; MedDiet categories were created: < 23 and ≥ 23, as per HNNHS adult population median score
7. BP: Blood Pressure

Significant between group differences were identified in age groups (p<0.05), with the majority of consumers over 60 years being grouped in the highest total SFA intake tertile, whereas the majority of those 45-59 years in the lowest total SFA intake tertile. Significant between group differences were also found in marital status, with more singles having higher SFA intake from SBGs (p<0.05), and in smoking status, with those categorized in the 3^rd^ tertile never having smoked (p<0.05 for between groups and trend). Meddiet score was significantly decreased with higher total SFA intake (p<0.001 for between groups and trend); a significant p-trend value was also observed in MedDiet categories (p<0.05). Finally, significant differences were found in sodium intake levels, with the majority of those categorized in the highest sodium intake category (≥ 2300 mg/day) also having the highest total SFA intakes (p<0.05 for between groups and trend). A significant p-trend (<0.05) was also found in hypertension status, with more of participants categorized as hypertensives also being categorized in the 3^rd^ tertile. No differences were found for age, sex, education level, employment status, physical activity status, mean SBP, mean DBP, dyslipidemia and CVD status.

**Figure S1:** Palmitic acid intakes, as a percentage of daily total energy intake at median (p25, p75)^a^ by SBG^b^ product in 2015 and 2021, using substitution models^c^

1. p25, p75: 25^th^, 75^th^ percentiles
2. SBG: Savory baked goods
3. Substitution models: measured content in savory baked goods in 2021 replaced those already used in previous research during the Hellenic National Nutrition and Health Survey study years (2015) to evaluate palmitic acid intake amount post Regulation (EU) 2019/649, assuming intakes of other foods remained constant.
